# Seasonality of main childhood embryonal tumours and rhabdomyosarcoma, France, 2000–2015

**DOI:** 10.1002/cam4.5624

**Published:** 2023-02-01

**Authors:** Danielle Awounou, Brigitte Lacour, Emmanuel Desandes, Sandra Guissou, Nathalie Cassoux, François Doz, Christelle Dufour, Véronique Minard‐Colin, Gudrun Schleiermacher, Sophie Taque, Arnauld Verschuur, Jacqueline Clavel, Stéphanie Goujon

**Affiliations:** ^1^ Inserm, UMR 1153 Centre of Research in Epidemiology and StatisticS (CRESS) Epidemiology of childhood and adolescent cancers team (EPICEA) Villejuif France; ^2^ Université Paris Cité Paris France; ^3^ French National Registry of Childhood Solid Tumours (RNTSE) CHU Nancy Nancy France; ^4^ Department of Surgical Oncology Institut Curie Paris France; ^5^ SIREDO Centre (Care, Innovation, Research In Pediatric, Adolescent and Young Adult Oncology) Institut Curie Paris France; ^6^ Department of Paediatric and Adolescent Oncology, Institut Gustave Roussy Université Paris Saclay Villejuif France; ^7^ Inserm, UMR 1015 Université Paris Saclay Villejuif France; ^8^ Department of Paediatrics CHU Rennes Rennes France; ^9^ Department of Paediatric Haematology, Immunology and Oncology Children Hospital of La Timone, APHM Marseille France; ^10^ French National Registry of Childhood Haematological Malignancies (RNHE) Villejuif France

**Keywords:** embryonal tumour, month of birth, month of diagnosis, population‐based cancer registry, seasonality

## Abstract

Few studies have investigated the seasonal patterns of embryonal tumours. Based on data from the French National Registry of Childhood Cancers, the present study aimed to investigate seasonal variations in embryonal tumour incidence rates by month of birth and by month of diagnosis. The study included 6635 primary embryonal tumour cases diagnosed before the age of 15 years over the period 2000‐2015 in mainland France. Assuming monthly variations in incidence rates were homogeneous over 2000‐2015, we used a Poisson regression model to test for overall heterogeneity in standardised incidence ratios (SIRs) by month of birth or diagnosis. The seasonal scan statistic method was used to detect monthly excesses or deficits of embryonal tumour cases over the whole study period. The annual reproducibility of the observed monthly variations was formally tested. An overall heterogeneity in incidence rates by month of birth was observed for rhabdomyosarcoma in boys only. Based on the month of diagnosis, a seasonality was evidenced for unilateral retinoblastoma, with a lower incidence rate in the summer (SIR_Jul‐Aug_ = 0.68, 95% CI = 0.52‐0.87), whilst the incidence rate of rhabdomyosarcoma tended to be lower in August (SIR_Aug_ = 0.68, 95% CI = 0.52‐0.89). No seasonality was detected for the other embryonal tumour groups by month of birth or month of diagnosis. This study is one of the largest to have investigated the seasonality of childhood embryonal tumours. The study showed a seasonal variation in the incidence rates by month of diagnosis for unilateral retinoblastoma and rhabdomyosarcoma. Our findings are likely to reflect a delay in consultation during the summer months. However, the role of seasonally varying environmental exposures cannot be ruled out.

## Graphical abstract

This study is an in‐depth investigation of seasonal variations in the incidence rates of paediatric embryonal tumours in France (2000‐2015). The results showed a seasonality by month of diagnosis for unilateral retinoblastoma and rhabdomyosarcoma cases, with a lower incidence rate in the summer months. Although our findings are likely to reflect delays in consultation with a specialist, we cannot rule out their being due to seasonally varying environmental exposures. No seasonality by month of birth was observed for the main embryonal tumour groups (neuroblastoma, nephroblastoma, medulloblastoma, rhabdomyosarcoma, retinoblastoma and hepatoblastoma) or specific subgroups considered.

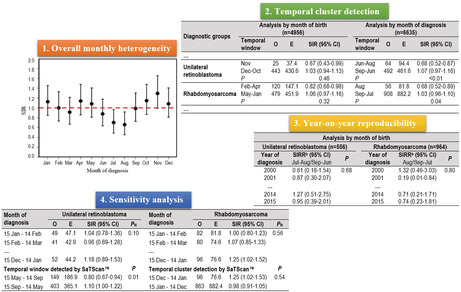

## 1 INTRODUCTION

Childhood embryonal tumours constitute a heterogeneous group of malignancies consisting of undifferentiated cells similar to those found in developing embryos.^1^ In France, those tumours account for roughly 25% of childhood cancers, with about 400 new cases per year. The main types of embryonal tumours are, in decreasing order of incidence, neuroblastoma, nephroblastoma (Wilms' tumour), medulloblastoma, retinoblastoma and hepatoblastoma.^2^ Rhabdomyosarcoma is the most common soft‐tissue sarcoma in children. As the ultrastructural features of the cells resemble primitive skeletal muscle‐lineage precursors, rhabdomyosarcoma is considered to be an embryonal tumour of skeletal muscle cell origin thought to derive from mesenchymal precursors.^3^, ^4^ Embryonal tumours occur more frequently at a young age, with half of the cases under 2 years old and over 80% less than 5 years old.^5^, ^6^ These key observations suggest that the tumorigenesis of those cancers might begin during the prenatal and/or perinatal periods, possibly due to errors in tissue growth or differentiation processes.^7^

Aetiological evidence remains poor for most embryonal tumours. The associations with certain genetic alterations have been well established (e.g. Beckwith‐Wiedemann syndrome and Wilms' tumour; *RB1* gene and retinoblastoma).^8^, ^9^, ^10^ However, overall, those factors only explain a small proportion of embryonal tumour cases.^11^ Thus, due to the early tumour onset, prenatal, perinatal and early postnatal exposures are suspected of playing a role in tumour development.^10^, ^12^, ^13^ In that context, seasonally‐varying environmental factors, such as infectious agents, traffic‐related air pollution and pesticides, have been reported to be potential risk factors for embryonal tumours in some studies.^14^, ^15^, ^16^, ^17^, ^18^, ^19^ To date, the evidence is somewhat limited, but if a seasonally‐varying factor were actually involved in embryonal tumour development, seasonal variations in tumour incidence rates might be observed. This issue has been little studied in the literature and the findings are rather inconsistent.^20^, ^21^, ^22^, ^23^, ^24^, ^25^, ^26^, ^27^ Undoubtedly, the rarity of the diseases makes it difficult to collect a sufficient number of cases to draw epidemiologically sound conclusions.

Based on data from the French National Registry of Childhood Cancers (RNCE),^28^ the present study aimed to investigate seasonal variations in embryonal tumour incidence rates by month of birth and month of diagnosis in the population of children living in mainland France during the period 2000‐2015. A large number of cases enabled us to evaluate seasonality for embryonal tumours by diagnostic group and main subgroup, separately. Our study is an exploratory study of seasonal variations in the incidence rate of embryonal tumours, which might provide etiological clues, but did not intend to point to any particular environmental risk factor.

## 2 MATERIALS AND METHODS

### 2.1 Embryonal tumour cases

All the cases aged 0‐14 years diagnosed with a primary embryonal tumour during the period 2000‐2015 and residing in mainland France at diagnosis were obtained from the RNCE. The Registry is a comprehensive database with three sources of information per case on average and contains precise information on diagnoses. Details of the registration process have been reported elsewhere.^29^ All the diagnoses were coded in compliance with the International Classification of Disease for Oncology, 3rd edition (ICDO‐3)^30^ and grouped as per the International Classification of Childhood Cancer, 3rd edition (ICCC‐3).^31^ The following diagnostic groups were analysed: medulloblastoma (ICCC‐3 IIIc.1), neuroblastoma (ICCC‐3 IVa, including ganglioneuroblastoma), retinoblastoma (ICCC‐3 V), nephroblastoma (ICCC‐3 VIa.1), hepatoblastoma (ICCC‐3 VIIa), and rhabdomyosarcoma (ICCC‐3 IXa).

In all, the study included 6635 embryonal tumour cases diagnosed between 1 January 2000 and 31 December 2015 and residing in mainland France at diagnosis. For the analyses by month of birth, we restricted the study to the 4956 embryonal tumour cases born during that period and living in mainland France at birth.

### 2.2 Population data

The population data were provided by the French Institute of Statistics and Economic Studies.

In the analyses by month of birth, the population at risk was estimated using birth data provided by month, year and gender. All the children born in France between 1 January 2000 and 31 December 2015 were included. The total number of live births in mainland France was, on average, 65 000 per month. Children were followed up until the earlier date of the day before their 15th birthday and 31 December 2015. The age‐specific person‐years at risk for the cohort of children born during month *m* of year *y* (${\mathrm{PY}}_{m,y,a}$) were calculated as follows (see Appendix S1 for an example):

$$\;{\mathrm{PY}}_{m,y,a}={\mathrm{NB}}_{m,y}*T_{m,y,a}$$

in which ${\mathrm{NB}}_{m,y}$ was the number of births during month *m* of year *y* and was assumed to be uniformly distributed throughout *m*. $T_{m,y,a}$, the time at‐risk (expressed in years) at age *a* years was estimated as follows: $T_{m,y,a}$=1 if *a* + *y* < 2015,

$$T_{m,y,a}=\frac{\left(12-m+0.5\right)}{12}$$

if *a* + *y* = 2015, 0 otherwise.

Census data were used for the analyses by month of diagnosis. The paediatric population was quite stable over the study period 2000‐2015, with about 11 million children aged less than 15 years living in mainland France each year. The age‐specific person‐years at risk were estimated as the average population of two consecutive years and weighted by the number of days in the month (see Appendix S2 for an example):

$${\mathrm{PY}}_{m,y,a}={\mathrm{PY}}_{y,a}*\frac{{\mathrm{ND}}_{m,y}}{{\mathrm{ND}}_{.,y}}\;,$$

with

$${\mathrm{PY}}_{y,a}=\frac{{\mathrm{POP}}_{y,a}+{\mathrm{POP}}_{\left(y+1\right),a}}{2}$$

in which ${\mathrm{PY}}_{m,y,a}$ is the person‐years for month *m* of year *y* and age *a* (*a* = 0 to 14 years); ${\mathrm{PY}}_{y,a}$, the total person‐years estimated for *y* and *a*; ${\mathrm{ND}}_{m,y}$, the number of days in month *m* of year *y*; ${\mathrm{ND}}_{.,y}$, the total number of days in year *y* (365 or 366 for leap years); ${\mathrm{POP}}_{y,a}$ and ${\mathrm{POP}}_{\left(y+1\right),a}$ are the numbers of children living in France and aged *a* years on 1 January of the years *y* and *y* + 1, respectively.

The number of embryonal tumours expected for each month of birth or diagnosis under the hypothesis of homogeneous incidence rates over the whole study period was then calculated by multiplying the age‐specific national incidence rates by the monthly person‐years at risk estimates.

National incidence rates by year of birth and year of diagnosis were stable over the period 2000‐2015 for all the embryonal tumour groups, except for medulloblastoma, for which the incidence rates for the years of birth were heterogeneous (Tables S1 and S2). For the latter group, sensitivity analysis by month of birth was conducted using annual age‐specific incidence rates as reference rates.

### 2.3 Statistical analyses

#### 2.3.1 Overall monthly heterogeneity

The standardised incidence ratio (SIR), defined as the ratio of the observed to the expected monthly numbers of cases, was used to assess the monthly variations in incidence rates over the study period. A Poisson regression model was fitted to the observed numbers of cases for month *m* and year *y* (*O*_m,y_) with the logarithm of the expected numbers of cases (*E*_m,y_) as an offset:

$$\ln\left(E\left(O_{m,y}\right)\right)=\ln\left(E_{m,y}\right)+\sum_{i=1}^{12}\beta_{i}*X_{i}\left(m\right)$$

$E\left(O_{m,y}\right)$ is the expectation value of the random variable $O_{m,y}$; $X_{i}\left(m\right)$ is the dummy variable with a value of one if *m* = *i*, 0 otherwise (*i* = 1 to 12); and $\beta_{i}$ is the parameter associated with month i, so that SIR_*i*_ = exp($\hat{\beta_{i}}$). A likelihood ratio test was used to test for an overall heterogeneity of the monthly SIRs. The analyses were performed using R (v.4.0.3) software.^32^

#### 2.3.2 Cluster detection

The seasonal scan statistic developed by M. Kulldorff^33^ was used to detect monthly excesses or deficits of embryonal tumour cases over the whole study period. In this method, the data are considered as a connecting loop, where 31 December is followed by 1 January. Using a moving window of varying size (≤6 months in this study), the method builds a set of cluster candidates composed of neighbouring months. For each candidate, the likelihood ratio was calculated based on the alternative hypothesis that the incidence rate within the window was different from the incidence rate outside it. The test statistic was defined as the maximum likelihood ratio over all cluster candidates; the associated window constituted the most likely cluster period. In this study, the statistical significance of the test statistic was estimated using 9999 Monte Carlo simulations^34^ carried out under the hypothesis of a Poisson distribution of the monthly number of cases and homogeneous monthly incidence rates over the whole study period. The seasonal scan method was implemented using SaTScan™ (v.9.7) software (SaTScan™ is a trademark of Martin Kulldorff. The SaTScan™ software was developed under the joint auspices of (1) Martin Kulldorff, (2) the National Cancer Institute and (3) Farzad Mostashari of the New York City Department of Health and Mental Hygiene).^35^

#### 2.3.3 Reproducibility analysis

Seasonal variations are temporal changes that recur from year to year. Where appropriate, we tested the year‐to‐year reproducibility of the monthly variations observed over the whole study period. To achieve this, we calculated the ratio of the SIR for the given cluster months to the SIR of the remaining months (SIRR), over the whole study period and for each year of birth or diagnosis separately. Between‐year heterogeneity of the SIRR was formally tested by introducing an interaction term between the cluster period and the year effects in the Poisson regression models.

#### 2.3.4 Additional analyses

Analyses of seasonality were carried out by gender for medulloblastoma and rhabdomyosarcoma cases, for which a male predominance has been well‐documented.^36^ Analysis by gender was not feasible for hepatoblastoma because of the limited number of cases. The seasonality of retinoblastoma was analysed by tumour laterality (unilateral or bilateral). Complementary analyses including stratification by age at diagnosis (≤18 vs. >18 months), tumour stage (localised vs. metastatic, in compliance with the International Neuroblastoma Staging System, INSS^37^) and *MYCN* gene amplification status (not amplified vs. amplified) were conducted for the patients with neuroblastoma because of the prognostic relevance of those factors. For the 230 (12.5%) and 332 (14.8%) neuroblastoma cases included in the analyses by month of birth and month of diagnosis, respectively, no information on *MYCN* status was available, mainly because no molecular analysis was done. We verified that those cases did not differ from the cases with known *MYCN* status in terms of distribution by month of birth or month of diagnosis (Table S3).

We conducted a sensitivity analysis with twelve 30‐day periods using the midpoint of consecutive months as cut‐offs (e.g. 15 January to 14 February, 15 February to 14 March, etc.), instead of the calendar months.

The significance level for all the tests was 0.05.

## 3 RESULTS

### 3.1 Case description

A total of 6635 cases aged less than 15 years and residing in mainland France were diagnosed with an embryonal tumour during the period 2000‐2015. Neuroblastoma was the most common embryonal tumour (2247 cases), followed by nephroblastoma (1412 cases), medulloblastoma (967 cases), rhabdomyosarcoma (964 cases), retinoblastoma (804 cases, 69% unilateral) and hepatoblastoma (241 cases) (Table 1). Forty per cent of the cases were diagnosed before age 2 years with neuroblastoma (47%), retinoblastoma (21%), and nephroblastoma (16%) the most common malignancies in that age group. About half of the neuroblastomas were metastatic (47.4%); 19.6% of the cases with known *MYCN* status (1915 cases) were *MYCN* amplified.

**TABLE 1:** Distribution of the embryonal tumour cases included in the analyses by month of diagnosis, and by diagnostic group, gender and age—mainland France, 2000‐2015

	Total			Gender					Age at diagnosis									
				boys		girls		sex ratio	<1 year		1 year		2 years		3‐4 years		≥5 years	
Diagnosis	n	%	IR	n	%	n	%		n	%	n	%	n	%	n	%	n	%
Neuroblastoma	2247	33.9	12.3	1189	52.9	1058	47.1	1.1	876	39.0	395	17.6	321	14.3	346	15.4	309	13.8
Localised	1182	52.6	6.5	609	51.5	573	48.5	1.0	545	46.1	223	18.9	121	10.2	146	12.4	147	12.4
Metastatic	1065	47.4	5.8	580	54.5	485	45.5	1.1	331	31.1	172	16.2	200	18.8	200	18.8	162	15.2
*MYCN* not amplified	1540	68.5	8.4	791	51.4	749	48.6	1.0	684	44.4	228	14.8	191	12.4	234	15.2	203	13.2
*MYCN* amplified	375	16.7	2.0	221	58.9	154	41.1	1.4	62	16.5	117	31.2	97	25.9	63	16.8	36	9.6
Unknown *MYCN* status	332	14.8	1.8	177	53.3	155	46.7	1.1	130	39.2	50	15.1	33	9.9	49	14.8	70	21.1
Nephroblastoma	1412	21.3	7.7	661	46.8	751	53.2	0.8	197	14.0	238	16.9	241	17.1	418	29.6	318	22.5
Medulloblastoma	967	14.6	5.3	622	64.3	345	35.7	1.7	41	4.2	62	6.4	81	8.4	157	16.2	626	64.7
Rhabdomyosarcoma	964	14.5	5.3	598	62.0	366	38.0	1.6	62	6.4	108	11.2	98	10.2	220	22.8	476	49.4
Retinoblastoma	804	12.1	4.4	406	50.5	398	49.5	1.0	347	43.2	228	28.4	113	14.1	86	10.7	30	3.7
Unilateral	556	69.2	3.0	265	47.7	291	52.3	0.9	185	33.3	164	29.5	100	18.0	80	14.4	27	4.9
Bilateral	248	30.8	1.4	141	56.9	107	43.1	1.3	162	65.3	64	25.8	13	5.2	6	2.4	3	1.2
Hepatoblastoma	241	3.6	1.3	148	61.4	93	38.6	1.5	73	30.3	69	28.6	38	15.8	32	13.3	29	12.0
All embryonal tumours	6635	100	36.2	3624	54.6	3011	45.4	1.1	1596	24.1	1100	16.6	892	13.4	1259	19.0	1788	26.9

Abbreviations: IR, annual incidence rate (cases/million/year); n, total number of cases; sex ratio, male to female IR ratio.

In all, 54.6% of the cases were boys and 45.4% were girls, with a sex ratio of 1.1. Male predominance was more marked for medulloblastoma (sex ratio: 1.7), rhabdomyosarcoma (sex ratio: 1.6) and hepatoblastoma (sex ratio: 1.5). Conversely, nephroblastoma appeared to be slightly more frequent in girls (sex ratio: 0.8).

The distribution of the cases by diagnostic group, gender and age was quite similar for the 4956 cases born during the study period 2000‐2015 (Table S4).

### 3.2 Seasonal variations in embryonal tumour incidence rates by month of birth

**FIGURE 1:**
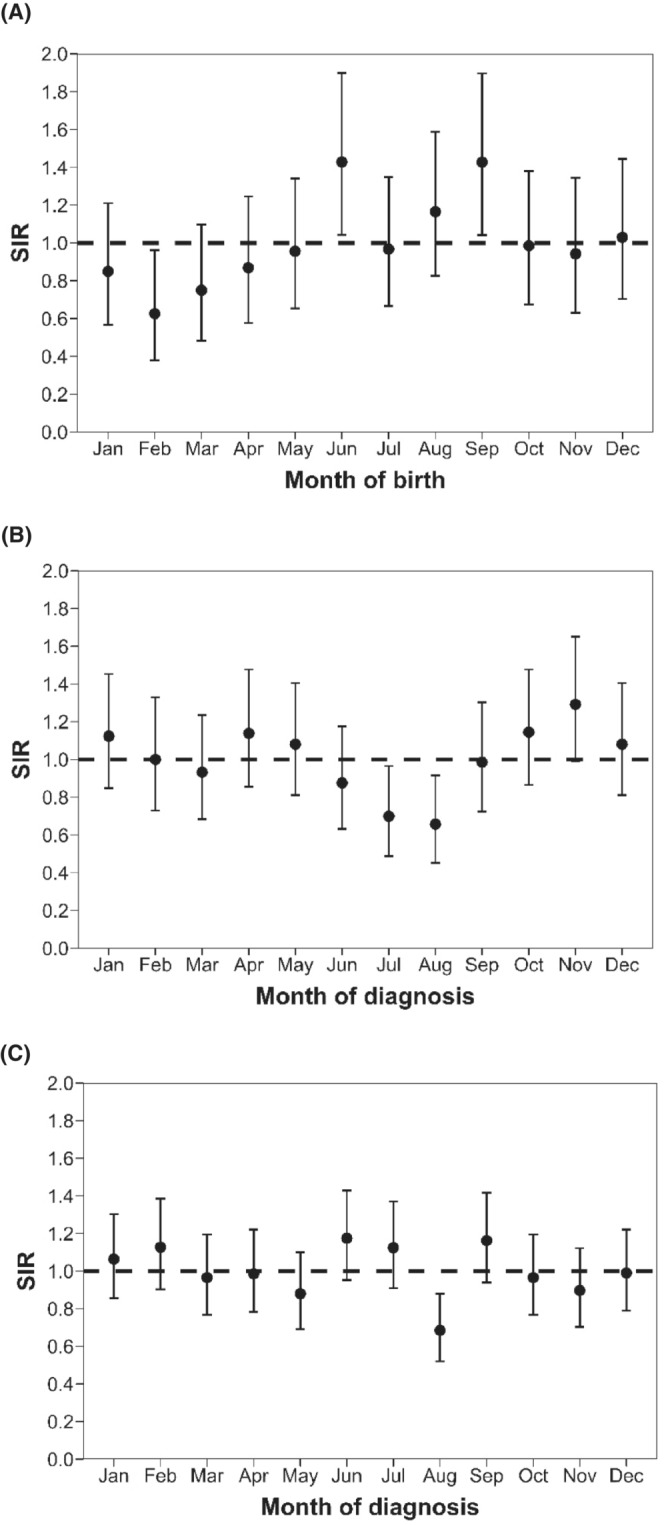
Monthly standardised incidence ratios (SIRs) with their 95% confidence intervals for the three situations where overall heterogeneity was detected—mainland France, RNCE, 2000‐2015. (A) SIR variations for rhabdomyosarcoma in boys by month of birth. (B) SIR variations for unilateral retinoblastoma by month of diagnosis. (C) SIR variations for rhabdomyosarcoma by month of diagnosis.

In the analyses by month of birth, no overall heterogeneity of the monthly SIRs was observed for the main groups of embryonal tumours (Table 2A) and no temporal cluster was detected using the SaTScan™ method (Table 3). The same results were observed for the following subgroup analyses: by age group, tumour stage and *MYCN* status for neuroblastoma; with stratification by gender for medulloblastoma; by considering laterality for retinoblastoma (Table 4). However, for rhabdomyosarcoma, an overall heterogeneity of monthly SIRs was suggested for boys (364 cases, *P*_*H*_ = 0.06) and the cluster analysis detected a four‐month birth period (June to September) associated with a higher incidence rate as compared to the remaining months (SIR_Jun‐Sep_ = 1.24, 95% CI = 1.05‐1.46 vs. SIR_Oct‐May_ = 0.88, 95% CI = 0.76‐1.00; Table 4). Although the annual numbers of cases were quite small (between 1.4 and 12.9 expected cases in the cluster window), the observed variations seemed reproducible from year to year, with a SIRR comparing June‐September to October‐May birth periods greater than one for 12 years out of 15 and no between‐year SIRR heterogeneity (*P* = 0.88; Table 5). Interestingly, the overall SIR was greater than one for each month of the cluster window, except July (SIR_Jul_ = 0.97, 95% CI = 0.67‐1.35; Figure 1A).

**TABLE 2:** Variations in embryonal tumour incidence rates by month of birth and month of diagnosis—mainland France, 2000‐2015

(A) Month of birth	Neuroblastoma (n = 1839)			Nephroblastoma (n = 1073)			Medulloblastoma (n = 541)			Rhabdomyosarcoma (n = 599)			Retinoblastoma (n = 698)			Hepatoblastoma (n = 206)		
	O	E	SIR (95% CI)	O	E	SIR (95% CI)	O	E	SIR (95% CI)	O	E	SIR (95% CI)	O	E	SIR (95% CI)	O	E	SIR (95% CI)
January	165	159.0	1.04 (0.89‐1.20)	74	93.2	0.79 (0.63‐0.99)	45	47.7	0.94 (0.69‐1.25)	53	52.4	1.01 (0.76‐1.31)	59	60.2	0.98 (0.75‐1.25)	23	17.8	1.29 (0.83‐1.90)
February	137	144.0	0.95 (0.80‐1.12)	94	84.4	1.11 (0.90‐1.35)	45	43.1	1.04 (0.77‐1.38)	37	47.4	0.78 (0.56‐1.06)	60	54.5	1.10 (0.84‐1.40)	18	16.1	1.12 (0.68‐1.71)
March	157	153.9	1.02 (0.87‐1.19)	95	90.1	1.05 (0.86‐1.28)	38	45.9	0.83 (0.59‐1.12)	40	50.6	0.79 (0.57‐1.06)	62	58.3	1.06 (0.82‐1.35)	20	17.2	1.16 (0.72‐1.75)
April	142	149.5	0.95 (0.80‐1.11)	88	87.6	1.00 (0.81‐1.23)	36	44.6	0.81 (0.57‐1.10)	43	49.1	0.88 (0.64‐1.16)	54	56.6	0.95 (0.72‐1.23)	19	16.7	1.13 (0.70‐1.72)
May	158	157.4	1.00 (0.86‐1.17)	91	92.1	0.99 (0.80‐1.21)	52	46.8	1.11 (0.84‐1.44)	55	51.6	1.07 (0.81‐1.37)	51	59.6	0.86 (0.64‐1.11)	19	17.6	1.08 (0.66‐1.64)
June	163	151.7	1.07 (0.92‐1.25)	86	88.6	0.97 (0.78‐1.19)	37	44.7	0.83 (0.59‐1.12)	59	49.5	1.19 (0.91‐1.52)	60	57.6	1.04 (0.80‐1.33)	16	17.0	0.94 (0.55‐1.48)
July	143	161.6	0.88 (0.75‐1.04)	96	94.3	1.02 (0.83‐1.24)	49	47.5	1.03 (0.77‐1.35)	51	52.6	0.97 (0.73‐1.26)	66	61.3	1.08 (0.84‐1.36)	16	18.1	0.88 (0.52‐1.39)
August	149	156.9	0.95 (0.81‐1.11)	98	91.3	1.07 (0.87‐1.30)	51	45.8	1.11 (0.84‐1.45)	56	50.9	1.10 (0.84‐1.42)	62	59.6	1.04 (0.80‐1.32)	14	17.6	0.80 (0.45‐1.29)
September	147	153.3	0.96 (0.81‐1.12)	96	89.1	1.08 (0.88‐1.31)	48	44.5	1.08 (0.80‐1.41)	60	49.5	1.21 (0.93‐1.54)	60	58.3	1.03 (0.79‐1.31)	14	17.2	0.82 (0.46‐1.32)
October	187	155.7	1.20 (1.04‐1.38)	88	90.5	0.97 (0.78‐1.19)	44	45.1	0.98 (0.71‐1.29)	48	50.3	0.96 (0.71‐1.25)	68	59.2	1.15 (0.90‐1.44)	19	17.4	1.09 (0.67‐1.65)
November	145	146.6	0.99 (0.84‐1.16)	81	85.1	0.95 (0.76‐1.17)	47	42.3	1.11 (0.82‐1.46)	50	47.2	1.06 (0.79‐1.38)	38	55.8	0.68 (0.49‐0.92)	13	16.4	0.79 (0.44‐1.30)
December	146	149.5	0.98 (0.83‐1.14)	86	86.7	0.99 (0.80‐1.22)	49	43.0	1.14 (0.85‐1.49)	47	48.0	0.98 (0.72‐1.29)	58	56.9	1.02 (0.78‐1.30)	15	16.8	0.89 (0.51‐1.43)
*P*_H_			0.46			0.80			0.74			0.48			0.49			0.92

(B) Month of diagnosis	Neuroblastoma (n = 2247)			Nephroblastoma (n = 1412)			Medulloblastoma (n = 967)			Rhabdomyosarcoma (n = 964)			Retinoblastoma (n = 804)			Hepatoblastoma (n = 241)		
	O	E	SIR (95% CI)	O	E	SIR (95% CI)	O	E	SIR (95% CI)	O	E	SIR (95% CI)	O	E	SIR (95% CI)	O	E	SIR (95% CI)
January	192	190.7	1.01 (0.87‐1.16)	103	119.8	0.86 (0.70‐1.04)	85	82.1	1.04 (0.83‐1.27)	87	81.8	1.06 (0.86‐1.30)	62	68.2	0.91 (0.70‐1.15)	24	20.5	1.17 (0.76‐1.71)
February	193	173.8	1.11 (0.96‐1.27)	111	109.2	1.02 (0.84‐1.22)	71	74.8	0.95 (0.75‐1.19)	84	74.6	1.13 (0.90‐1.39)	64	62.2	1.03 (0.80‐1.30)	21	18.6	1.13 (0.71‐1.68)
March	188	190.7	0.99 (0.85‐1.13)	117	119.8	0.98 (0.81‐1.16)	73	82.1	0.89 (0.70‐1.11)	79	81.8	0.97 (0.77‐1.19)	71	68.2	1.04 (0.82‐1.30)	27	20.5	1.32 (0.88‐1.88)
April	187	184.6	1.01 (0.87‐1.17)	114	116.0	0.98 (0.81‐1.17)	78	79.4	0.98 (0.78‐1.22)	78	79.2	0.99 (0.78‐1.22)	77	66.0	1.17 (0.92‐1.45)	26	19.8	1.31 (0.87‐1.88)
May	196	190.7	1.03 (0.89‐1.18)	126	119.8	1.05 (0.88‐1.25)	87	82.1	1.06 (0.85‐1.30)	72	81.8	0.88 (0.69‐1.10)	72	68.2	1.06 (0.83‐1.32)	17	20.5	0.83 (0.50‐1.29)
June	195	184.6	1.06 (0.92‐1.21)	122	116.0	1.05 (0.88‐1.25)	70	79.4	0.88 (0.69‐1.10)	93	79.2	1.17 (0.95‐1.43)	53	66.0	0.80 (0.61‐1.04)	20	19.8	1.01 (0.63‐1.52)
July	187	190.7	0.98 (0.85‐1.13)	120	119.8	1.00 (0.83‐1.19)	84	82.1	1.02 (0.82‐1.26)	92	81.8	1.12 (0.91‐1.37)	50	68.2	0.73 (0.55‐0.96)	16	20.5	0.78 (0.46‐1.23)
August	177	190.7	0.93 (0.80‐1.07)	102	119.8	0.85 (0.70‐1.03)	75	82.1	0.91 (0.72‐1.14)	56	81.8	0.68 (0.52‐0.88)	49	68.2	0.72 (0.54‐0.94)	18	20.5	0.88 (0.53‐1.35)
September	177	184.6	0.96 (0.82‐1.11)	116	116.0	1.00 (0.83‐1.19)	78	79.4	0.98 (0.78‐1.22)	92	79.2	1.16 (0.94‐1.42)	70	66.0	1.06 (0.83‐1.33)	17	19.8	0.86 (0.51‐1.33)
October	176	190.7	0.92 (0.79‐1.07)	129	119.8	1.08 (0.90‐1.27)	91	82.1	1.11 (0.90‐1.35)	79	81.8	0.97 (0.77‐1.19)	80	68.2	1.17 (0.93‐1.45)	25	20.5	1.22 (0.80‐1.77)
November	191	184.6	1.03 (0.89‐1.19)	144	116.0	1.24 (1.05‐1.46)	87	79.4	1.10 (0.88‐1.34)	71	79.2	0.90 (0.70‐1.12)	78	66.0	1.18 (0.94‐1.46)	13	19.8	0.66 (0.36‐1.08)
December	188	190.7	0.99 (0.85‐1.13)	108	119.8	0.90 (0.74‐1.08)	88	82.1	1.07 (0.86‐1.31)	81	81.8	0.99 (0.79‐1.22)	78	68.2	1.14 (0.91‐1.42)	17	20.5	0.83 (0.50‐1.29)
*P*_H_			0.89			0.22			0.90			0.07			0.02			0.43

Abbreviations: 95% CI, 95% confidence interval; n, total number of cases; O and E, monthly observed and expected number of cases; *P*_H_, statistical significance threshold (P‐value) of the likelihood ratio test for the overall heterogeneity of monthly SIRs; SIR, standardised incidence ratio.

**TABLE 3:** Temporal cluster detection analyses for the main diagnostic groups of embryonal tumours—mainland France, 2000‐2015

Main diagnostic groups	Analysis by month of birth (n = 4956)				Analysis by month of diagnosis (n = 6635)			
	Temporal window	O	E	SIR (95% CI)	Temporal window	O	E	SIR (95% CI)
Neuroblastoma	**Oct**	**187**	**155.7**	**1.20 (1.04‐1.39)**	**Feb**	**193**	**173.8**	**1.11 (0.96‐1.28)**
	Nov‐Sep	1652	1683.3	0.98 (0.93‐1.03)	Mar‐Jan	2054	2073.2	0.99 (0.95‐1.03)
	*P*			0.30	*P*			0.47
Nephroblastoma	**Jan**	**74**	**93.2**	**0.79 (0.62‐1.00)**	**Nov**	**144**	**116.0**	**1.24 (1.05‐1.46)**
	Feb‐Dec	999	979.8	1.02 (0.96‐1.08)	Dec‐Oct	1268	1296.0	0.98 (0.93‐1.03)
	*P*			0.46	*P*			.18
Medulloblastoma	**Mar‐Apr**	**74**	**90.6**	**0.82 (0.64‐1.03)**	**Mar‐Aug**	**467**	**487.1**	**0.96 (0.87‐1.05)**
	May‐Feb	467	450.4	1.04 (0.94‐1.14)	Sep‐Feb	500	479.9	1.04 (0.95‐1.14)
	*P*			0.63	*P*			0.92
Rhabdomyosarcoma	**Feb‐Apr**	**120**	**147.1**	**0.82 (0.68‐0.98)**	**Aug**	**56**	**81.8**	**0.68 (0.52‐0.89)**
	May‐Jan	479	451.9	1.06 (0.97‐1.16)	Sep‐Jul	908	882.2	1.03 (0.96‐1.10)
	*P*			0.32	*P*			0.04
Retinoblastoma	**Nov**	**38**	**55.8**	**0.68 (0.48‐0.93)**	**Jun‐Aug**	**152**	**202.5**	**0.75 (0.64‐0.88)**
	Dec‐Oct	660	642.2	1.03 (0.95‐1.11)	Sep‐May	652	601.5	1.08 (1.00‐1.17)
	*P*			0.22	*P*			<0.01
Hepatoblastoma	**Jan‐May**	**99**	**85.5**	**1.16 (0.94‐1.41)**	**Jan‐Apr**	**98**	**79.3**	1.24 (1.00‐1.51)
	Jun‐Dec	107	120.5	0.89 (0.73‐1.07)	May‐Dec	143	161.7	0.88 (0.75‐1.04)
	*P*			0.63	*P*			0.21

Abbreviations: 95% CI, 95% confidence interval; n, total number of cases; O and E, monthly observed and expected number of cases; *P*, *P*‐value for the most likely cluster estimated with 9999 Monte Carlo simulations (SaTScan™); SIR, standardised incidence ratio. Bold lines indicate the most likely cluster window detected by the seasonal scan method (SaTScan™).

**TABLE 4:** Subgroup analyses of seasonal variation in incidence rates of embryonal tumour—mainland France, 2000‐2015

Case characteristics	n (%)	*P*_H_	Most likely cluster window	O	E	SIR (95% CI)	*P*
Analysis by month of birth							
Neuroblastoma							
≤18 months at diagnosis	1056 (57.4%)	0.62	Jul‐Sep	242	271.7	0.89 (0.78‐1.01)	0.45
>18 months at diagnosis	783 (42.6%)	0.31	Sep‐Nov	226	193.0	1.17 (1.02‐1.33)	0.17
Localised (stages 1, 2 and 3)	996 (54.2%)	0.41	Jul‐Sep	227	255.6	0.89 (0.78‐1.01)	0.46
Metastatic (stages 4 and 4S)	843 (45.8%)	0.79	Oct	84	71.3	1.18 (0.94‐1.46)	.92
*MYCN* not amplified	1307 (81.2%)	0.72	Apr	92	106.2	0.87 (0.70‐1.06)	0.93
*MYCN* amplified	302 (18.8%)	0.94	Oct	34	25.6	1.33 (0.92‐1.86)	0.85
Unknown *MYCN* status	230						
Medulloblastoma							
Males	356 (65.8%)	0.44	Jul‐Dec	199	176.4	1.13 (0.98‐1.30)	0.40
Females	185 (34.2%)	0.77	Dec‐Feb	55	45.8	1.20 (0.90‐1.56)	0.86
Rhabdomyosarcoma							
Males	364 (60.8%)	0.06	Jun‐Sep	153	123.2	1.24 (1.05‐1.46)	0.05
Females	235 (39.2%)	0.91	Nov‐Feb	85	76.6	1.11 (0.89‐1.37)	0.98
Retinoblastoma							
Unilateral	468 (67.0%)	0.16	Nov	25	37.4	0.67 (0.43‐0.99)	0.46
Bilateral	230 (33.0%)	0.67	Jul‐Aug	51	39.9	1.28 (0.95‐1.68)	0.77
Analysis by month of diagnosis							
Neuroblastoma							
≤18 months at diagnosis	1138 (50.6%)	0.65	Jan‐Apr	408	374.7	1.09 (0.99‐1.20)	0.36
>18 months at diagnosis	1109 (49.4%)	0.27	Nov	113	91.1	1.24 (1.02‐1.49)	0.34
Localised (stages 1, 2 and 3)	1182 (52.6%)	0.94	Jul‐Dec	559	595.5	0.94 (0.86‐1.02)	0.63
Metastatic (stages 4 and 4S)	1065 (47.4%)	0.66	Feb	98	82.4	1.19 (0.97‐1.45)	0.44
*MYCN* not amplified	1540 (80.4%)	0.86	Feb	138	119.1	1.16 (0.97‐1.37)	0.37
*MYCN* amplified	375 (19.6%)	0.91	Oct‐Nov	75	62.6	1.20 (0.94‐1.50)	0.84
Unknown *MYCN* status	332						
Medulloblastoma							
Males	622 (64.3%)	0.19	Mar‐Aug	282	313.3	0.90 (0.80‐1.01)	0.21
Females	345 (35.7%)	0.66	Sep‐Oct	45	57.6	0.78 (0.57‐1.05)	0.70
Rhabdomyosarcoma							
Males	598 (62.0%)	0.34	Aug	35	50.8	0.69 (0.48‐0.96)	0.26
Females	366 (38.0%)	0.23	Dec‐Feb	105	90.5	1.16 (0.95‐1.40)	0.54
Retinoblastoma							
Unilateral	556 (69.2%)	0.07	Jul‐Aug	64	94.4	0.68 (0.52‐0.87)	<0.01
Bilateral	248 (30.8%)	0.05	Jan	9	21.0	0.43 (0.20‐0.81)	0.07

Abbreviations: 95% CI, 95% confidence interval; n, total number of cases; O and E, monthly observed and expected number of cases; *P*_H_, *P*‐value of the likelihood ratio test for the overall heterogeneity of monthly SIRs; *P*, *P*‐value for the most likely cluster estimated with 9999 Monte Carlo simulations (SaTScan™); SIR, standardised incidence ratio.

**TABLE 5:** Year‐on‐year reproducibility of the monthly variations observed over the whole study period—mainland France, 2000‐2015

Analysis by month of birth			Analysis by month of diagnosis					
Rhabdomyosarcoma in boys (n = 364)			Unilateral retinoblastoma (n = 556)			Rhabdomyosarcoma (n = 599)		
Year of birth	**SIRR** ^a^ **(95% CI)** Jun‐Sep/Oct‐May	*P*	Year of diagnosis	**SIRR** ^b^ **(95% CI)** Jul‐Aug/Sep‐Jun	*P*	Year of diagnosis	**SIRR** ^c^ **(95% CI)** Aug/Sep‐Jul	P
2000	1.20 (0.59‐2.37)	0.88	2000	0.61 (0.18‐1.54)	0.68	2000	1.32 (0.46‐3.03)	0.80
2001	1.02 (0.52‐1.91)		2001	0.87 (0.30‐2.07)		2001	0.19 (0.01‐0.84)	
2002	2.33 (1.18‐4.69)		2002	0.87 (0.30‐2.07)		2002	0.76 (0.23‐1.84)	
2003	1.45 (0.71‐2.88)		2003	0.61 (0.18‐1.54)		2003	0.66 (0.20‐1.60)	
2004	2.03 (1.08‐3.84)		2004	0.38 (0.06‐1.26)		2004	0.42 (0.07‐1.36)	
2005	1.66 (0.76‐3.60)		2005	0.29 (0.05‐0.94)		2005	0.51 (0.13‐1.38)	
2006	1.00 (0.47‐2.04)		2006	0.95 (0.39‐2.01)		2006	0.19 (0.01‐0.87)	
2007	1.54 (0.68‐3.38)		2007	0.35 (0.06‐1.16)		2007	1.06 (0.41‐2.26)	
2008	0.86 (0.33‐2.01)		2008	0.77 (0.29‐1.70)		2008	0.58 (0.14‐1.57)	
2009	1.13 (0.42‐2.80)		2009	0.47 (0.11‐1.32)		2009	1.06 (0.37‐2.40)	
2010	1.43 (0.55‐3.53)		2010	0.16 (0.01‐0.76)		2010	0.91 (0.32‐2.06)	
2011	1.95 (0.67‐5.69)		2011	0.61 (0.15‐1.75)		2011	0.54 (0.13‐1.45)	
2012	1.58 (0.56‐4.23)		2012	0.33 (0.05‐1.08)		2012	0.43 (0.07‐1.39)	
2013	0.80 (0.11‐3.71)		2013	0.61 (0.18‐1.54)		2013	0.71 (0.21‐1.71)	
2014	2.16 (0.09‐54.6)		2014	1.27 (0.51‐2.75)		2014	0.71 (0.21‐1.71)	
2015	‐		2015	0.95 (0.39‐2.01)		2015	0.74 (0.23‐1.81)	

Abbreviations: 95% CI, 95% confidence interval; n, total number of cases; *P*, *P*‐value of the likelihood ratio test for the overall heterogeneity of SIRRs; SIRR, ratio of the standardised incidence ratio (SIR).

^a^ Ratio of the SIRs of the June‐September and October‐May birth periods.

^b^ Ratio of the SIRs of the July‐August and September‐June periods of diagnosis.

^c^ Ratio of the SIRs for August and September‐July periods of diagnosis.

In the sensitivity analysis based on mid‐month breakpoints, the window detected by the scan method extended from mid‐December to mid‐April with a lower number of cases than expected (SIR = 0.78, 95% CI = 0.62‐0.95; Table S5). Symmetrically, the SIR was greater than 1 for the mid‐April to mid‐December birth period.

The results for medulloblastoma were unchanged when we considered annual age‐specific incidence rates as reference rates (Table S6).

### 3.3 Seasonal variations in embryonal tumour incidence rates by month of diagnosis

Analyses by month of diagnosis showed statistically significant heterogeneity of the monthly SIRs for retinoblastoma (*P* = 0.02; Table 2B), with a time window from June to August associated with a lower incidence rate in comparison to the September‐May period in the temporal cluster analysis (SIR_Jun‐Aug_ = 0.75, 95% CI = 0.64‐0.88 vs. SIR_Sep‐May_ = 1.08, 95% CI = 1.00‐1.17; Table 3). Those variations were reproducible from year to year (*P* = 0.93; not shown). Similar seasonal variations were observed for unilateral retinoblastoma (69% of the retinoblastoma cases) with a lower incidence rate in July‐August period (SIR_Jul‐Aug_ = 0.68, 95% CI = 0.52‐0.87; Table 4, Table 5 and Figure 1B). The analyses of bilateral retinoblastoma cases showed an overall heterogeneity of the monthly SIRs with a lower incidence rate detected in January (SIR_Jan_ = 0.43, 95% CI = 0.20‐0.81; Table 4). The variations were of borderline significance and based on small numbers of cases (21 expected cases in January).

An overall heterogeneity and a lower incidence rate in August were also observed for rhabdomyosarcoma (SIR_Aug_ = 0.68, 95% CI = 0.52‐0.89; Table 2B, Table 3 and Figure 1C), and the variations were reproducible year on year (*P* = 0.80; Table 5). No monthly variation was observed for the other embryonal tumour groups (Table 2B, Table 3 and Table 4).

The sensitivity analyses with twelve 30‐day periods defined by spanning consecutive months confirmed the seasonality of unilateral retinoblastoma with, however, a larger detected temporal window than in the main analyses (from mid‐May to mid‐September SIR = 0.78, 95% CI = 0.66‐0.92; Table S5). No seasonality was found for bilateral retinoblastoma or rhabdomyosarcoma (not shown).

## 4 DISCUSSION

This study is an in‐depth investigation of seasonal variations in the incidence rates of paediatric embryonal tumours in France over the period 2000‐2015. Regarding the time of birth, seasonal variations were suggested for rhabdomyosarcoma, with a higher incidence rate in boys born during the June‐August period and, symmetrically, a lower incidence rate for boys born during the September‐May period. Regarding the time of diagnosis, seasonal variations were evidenced for unilateral retinoblastoma, with a lower incidence rate for the summer period, and rhabdomyosarcoma, with a lower incidence rate in August. No seasonality was observed for the other embryonal tumour groups or specific subgroups considered.

For neuroblastoma, the most common embryonal tumour, there was no evidence of seasonal variation in incidence rates by month of birth or diagnosis. These results are consistent with those of three studies,^21^, ^24^, ^25^ of which a large US study included about 1500 neuroblastoma cases.^21^ In neuroblastoma, patients younger than 18 months (median age neuroblastoma diagnosis) have a greater overall survival rate than patients older than 18 months.^38^, ^39^ Approximately half of the children diagnosed with neuroblastoma have metastatic disease at diagnosis and, except for infants, have a low survival probability.^40^ An amplification of the *MYCN* oncogene (encoding a transcription factor), is also associated with a poor survival rate, even in localised disease or in infants.^6^, ^39^, ^41^ Stratifying by those factors did not reveal seasonal variations. A large Italian neuroblastoma cohort study (2756 cases) also investigated the seasonal pattern of neuroblastoma incidence rate by month of birth and diagnosis by carrying out subgroup analyses by age (0‐11, 12‐59 and ≥60 months), gender and tumour stage (localised, including stages 1, 2 and 3; stage 4 and stage 4S as defined in the INSS). No statistically significant variation was reported, except for stage 4S neuroblastoma cases, which was in excess amongst children born during the summer months.^27^ No excess was observed in our study.

We did not observe any statistically significant seasonal variations by month of birth or month of diagnosis in the incidence rate of the most common type of malignant childhood brain tumour, medulloblastoma, either overall or in the analyses by gender. In the literature, investigations for the seasonality of medulloblastoma by month of birth have yielded inconsistent results.^22^, ^23^, ^25^, ^26^ Halperin et *al* ^22^ analysed three different US datasets and found that medulloblastoma cases in North Carolina were born in the fall more frequently than expected, whilst no seasonality was observed with the Californian (Los Angeles and San Jose/Monterey regions) or national Surveillance, Epidemiology, and End Result (SEER) datasets. Another US study, which used the Central Brain Tumour Registry of the United States (CBTRUS) data, reported a seasonal variation for the subgroup of medulloblastoma, not otherwise specified, with a higher rate for children born in October.^23^ These variations were observed, in particular, for children aged 5‐19 years at diagnosis and for females. Consistently with our results, no evidence of seasonal variation in medulloblastoma incidence rates by month of birth was found in two studies conducted in the North of England^25^ and Denmark,^26^ respectively. To our knowledge, only one study has addressed the seasonality of central nervous system embryonal tumours by month of diagnosis; it did not find any seasonal variation.^25^

No seasonal patterns based on the month of birth or diagnosis were found for nephroblastoma or hepatoblastoma. Few studies have explored the seasonality of those types of embryonal tumours. The results of the Northern England study of childhood cancer were similar to ours with no evidence of seasonality in renal tumour incidence by month of birth or diagnosis.^25^ Hepatoblastomas were excluded from the subgroup analyses in the Northern England study because they accounted for less than 20 cases over the 37‐year study period. In a US study of 228 cases, there was a significant seasonal variation in hepatoblastoma incidence rates based on the month of diagnosis, with a higher rate in summer.^21^

In this study, we observed seasonal variations in the incidence rates of rhabdomyosarcoma in boys by month of birth, with a higher incidence rate in the June‐September birth period, but not for all rhabdomyosarcomas. This finding should be interpreted with caution as the SIR was close to one in July and thus the monthly incidence rates were not homogeneous over the detected window. In addition, the window detected by the cluster analysis was quite different in the sensitivity analysis based on mid‐month cut‐offs. Overall, these results suggest an overall heterogeneity in incidence rates by month of birth rather than seasonality. Although there is no clear‐cut seasonality, but rather an overall heterogeneity of rhabdomyosarcoma incidence rates in boys only, gender differences in disease incidence may provide important clues to disease pathogenesis and further research on this subject is to be encouraged. A lower incidence rate was also observed for rhabdomyosarcoma in August in the analysis by month of diagnosis. This result did not persist in the sensitivity analyses, which may be interpreted as a lack of robustness of the main result or as an indication that the period of lower incidence rate was strictly limited to August. The study conducted in the UK found no seasonal pattern of rhabdomyosarcoma incidence rates by month of birth or diagnosis, overall, or for males and females, separately.^25^ In contrast, in the US study, which included a larger number of rhabdomyosarcoma cases (861 cases, versus 114 in Basta *et al* ^25^), Ross *et al* ^21^ reported a seasonal variation in rhabdomyosarcoma incidence, with a higher rate in spring/summer, which is not concordant with the lower incidence rate we observed in August.

Retinoblastoma and in particular unilateral retinoblastoma also showed marked seasonal variations in the months of diagnosis, with a lower incidence rate during the summer, but no variations in the months of birth. Two studies, one conducted in the UK (88 retinoblastoma cases) and the other in Japan (753 sporadic unilateral retinoblastoma cases), investigated variations in retinoblastoma cases by month of birth or diagnosis. No seasonality was detected.^20^, ^25^ The fact that some studies have reported a higher incidence rate of sporadic retinoblastoma in developing countries than in developed countries suggests that environmental and behavioural factors associated with low socio‐economic status may play a role in the aetiopathogenesis of retinoblastoma.^42^, ^43^ In light of the results of several case studies, human papillomavirus (HPV) is suspected of being a risk factor for sporadic retinoblastoma.^42^, ^44^, ^45^, ^46^ In addition, some studies have suggested a possible role of exposure to pesticides and air pollution in the development of childhood retinoblastoma.^16^, ^47^, ^48^ Since there is a two‐hit hypothesis for the aetiology of retinoblastoma,^49^ exposures to infections or other environmental factors may be associated with both somatic events leading to sporadic retinoblastoma, as proposed by Bunin *et al*.^50^ Although the involvement of an environmental factor in the seasonality we observed cannot be ruled out, our findings are more likely to reflect a delay in consulting specialists possibly due to delayed parental care‐seeking and reduced access to consultations during the summer. In France, workers are entitled to 5 weeks annual paid vacation^51^ and most people tend to be on holiday in July/August. In addition, leucocoria is the most frequent clinical sign of retinoblastoma that prompts parents to consult a specialist.^52^, ^53^, ^54^ Leucocoria is mainly detected in dim light^52^, ^54^ and at certain angles, and may thus be less readily detected in summer, which may also delay consultation of a specialist.

In a Swedish epidemiological study of adult cancers, a lower number of melanomas, and prostate, breast and thyroid cancers was also observed during the summer.^55^ A clinical feature common to those cancers and childhood retinoblastoma and rhabdomyosarcoma is that they often present with mild, non‐acute symptoms, which may be an explanation for delayed self‐ or parental referral during the summer. The other embryonal tumours considered in the study may be associated with more intense clinical signs, leading to prompter consultation irrespective of the time of year. Potential delays in self‐referral are thus less likely to lead to seasonal variations in the incidence rates of those tumours.

Our study has certain limitations. First, the date of onset of the initial symptoms was not known. Although the interval between symptom onset and disease diagnosis may vary between children, the diagnosis date was the best available approximation for the disease onset date. Also, the registry does not include any information on race that could have been used as a surrogate for genetic predisposing factors. Another limitation is that the borders of the cluster detected by the scan method depend on the temporal units under consideration. The method can detect a temporal cluster associated with excesses or deficits overall but without there being necessarily an excess/deficit in all the units that make up the cluster. In consequence, we performed a sensitivity analysis, in which we considered twelve 30‐day periods straddling two consecutive months in order to enable the detection of temporal cluster periods that might not completely coincide with the calendar months, and evaluation of the robustness of the clusters detected in the main analyses. In addition, although our study is one of the largest to have investigated the seasonality of embryonal tumours, some subtypes could not be studied (e.g. molecular subgroups of medulloblastoma).

Despite its limitations, this study has several strengths, the major one being that it is based on a large and high‐quality population‐based registry, with nationwide coverage over a long recent time period. A recent systematic review of the seasonality of central nervous system tumours drew attention to the methodological weaknesses of the studies reported in the literature to date, including, in particular, a lack of statistical power, which prevents sub‐analyses by histological subtype.^56^ The limitation is also strongly applicable to studies of childhood embryonal tumours, because of the rarity of those diseases. However, the present study is one of the largest conducted and the number of cases included enabled us to investigate for potential seasonal variations in the incidence rates of the main types and subtypes of embryonal tumour by month of birth and month of diagnosis. It also enabled testing for the annualreproducibility of the observed monthly variations, which is an important criterion in the context of concluding that seasonality exists, an issue that is not addressed or discussed in most previous studies. A further strength of this study consists in the fact that two distinct complementary approaches were used with no a priori hypothesis as to a specific expected seasonal pattern. In contrast, other tests of seasonality usually assume a sinusoidal pattern with successive symmetrical peaks and troughs and a pre‐specified periodicity. The two approaches led to similar conclusions.

In conclusion, no seasonality was observed for embryonal tumour by month of birth. There was some evidence of seasonality by month of diagnosis for retinoblastoma and rhabdomyosarcoma cases, with a lower incidence rate in the summer months. Although these results are likely to reflect delays in consultation with a specialist, we cannot rule out their being due to seasonally varying environmental exposures.

## AUTHOR CONTRIBUTIONS

**Danielle Awounou:** Conceptualization (supporting); data curation (lead); formal analysis (lead); methodology (lead); visualization (lead); writing – original draft (lead); writing – review and editing (equal). **Brigitte Lacour:** Conceptualization (supporting); funding acquisition (equal); investigation (equal); methodology (supporting); writing – review and editing (equal). **Emmanuel Desandes:** Investigation (equal); methodology (supporting); writing – review and editing (equal). **Sandra Guissou:** Investigation (equal); writing – review and editing (equal). **Nathalie Cassoux:** Methodology (supporting); writing – review and editing (equal). **François Doz:** Methodology (supporting); writing – review and editing (equal). **Christelle Dufour:** Methodology (supporting); writing – review and editing (equal). **Veronique Minard:** Methodology (supporting); writing – review and editing (equal). **Gudrun Schleiermacher:** Methodology (supporting); writing – review and editing (equal). **Sophie Taque:** Methodology (supporting); writing – review and editing (equal). **Arnauld Verschuur:** Methodology (supporting); writing – review and editing (equal). **Jacqueline Clavel:** Conceptualization (lead); funding acquisition (equal); investigation (equal); methodology (lead); project administration (equal); supervision (supporting); visualization (supporting); writing – review and editing (equal). **Stéphanie Goujon:** Conceptualization (lead); data curation (supporting); methodology (lead); project administration (equal); supervision (lead); visualization (supporting); writing – review and editing (equal).

## FUNDING INFORMATION

This study was supported by grants from the ‘*Institut national de la santé et de la recherche médicale* (*INSERM*)’, the ‘*Institut national du cancer* (*INCa*)’ and ‘*Santé publique France*’. Danielle Awounou also received funding from the ‘*Institut national du cancer* (*INCa_15593*)’ for her PhD.

## CONFLICT OF INTEREST

The authors declare that they do not have any conflict of interest.

## ETHICAL APPROVAL AND CONSENT TO PARTICIPATE

The research undertaken with the RNCE data is covered by agreements on the ethical use of data and the protection of personal data and has been approved by the French national authorities (French data protection authority CNIL No. 900183). All patients were able to formulate their opposition before registration in the RNCE database.

## Supporting information


Appendix S1

## Data Availability

The datasets supporting the results of this study are stored in the Inserm EPICEA team facilities, in compliance with the general data protection regulation. Data are available from the authors upon reasonable request and with the permission of the Inserm EPICEA team.
