# Supplementary material for: Seasonality of main childhood embryonal tumours and rhabdomyosarcoma, France, 2000–2015
Source: Cancer Med. 2023 Feb 1;12(7):8789–803. doi: 10.1002/cam4.5624 (PMC10134357; doi:10.1002/cam4.5624)
Supplement: Supplementary file 1 — Appendix S1 [file CAM4-12-8789-s001.docx]

**Supplementary Information**

**Seasonality of main childhood embryonal tumours and rhabdomyosarcoma, France, 2000-2015**

Danielle Awounou, Brigitte Lacour, Emmanuel Desandes, Sandra Guissou, Nathalie Cassoux, François Doz, Christelle Dufour, Véronique Minard-Colin, Gudrun Schleiermacher, Sophie Taque, Arnauld Verschuur, Jacqueline Clavel, and Stéphanie Goujon

**Contents**

[**Appendix 1.** Formula for calculation of age-specific person-years at risk for the analyses by month of birth with an example…………………………………………………………..…….2](#_Toc117858511)

[**Appendix 2**. Formula for calculation of age-specific person-years at risk for the analyses by month of diagnosis with an example………………………………………………………….. 3](#_Toc117858512)

[**Table S1.** Variations in embryonal tumour incidence rates by year of birth – mainland France, 2000-2015 (source: RNCE)……………………………………………………………….…... 5](#_Toc117858513)

[**Table S2.** Variations in embryonal tumour incidence rates by year of diagnosis – mainland France, 2000-2015 (source: RNCE)…………………………………………………………... 6](#_Toc117858514)

[**Table S3.** Distribution of neuroblastoma cases by month of birth and month of diagnosis by *MYCN* status (known vs. unknown) - mainland France, 2000-2015 (source: RNCE)…….........7](#_Toc117858515)

[**Table S4.** Distribution of the embryonal tumour cases included in the analyses by month of birth, and by diagnostic group, gender and age - mainland France, 2000-2015 (source: RNCE)………………………………………………………………………………………… 8](#_Toc117858516)

[**Table S5.** Monthly variations based on twelve 30-day non-calendar month periods - mainland France, 2000-2015 (source: RNCE)…………………………………………………………... 9](#_Toc117858517)

[**Table S6.** Variations in medulloblastoma incidence rates by month of birth using annual age-specific incidence rates as reference rates - mainland France, 2000-2015 (source: RNCE)……………………………………………………………………………………..…10](#_Toc117858518)

# Appendix 1. Formula for calculation of age-specific person-years at risk for the analyses by month of birth with an example

The age-specific person-years at risk for the cohort of children born during month *m* of year *y* ($\mathrm{PY}_{m,y,a}$) were calculated as follows:

$\mathrm{PY}_{m,y,a}=\mathrm{NB}_{m,y}*T_{m,y,a}$

in which $\mathrm{NB}_{m,y}$ was the number of births during month *m* of year *y* and was assumed to be uniformly distributed throughout *m*. $T_{m,y,a}$, the time at-risk (expressed in years) at age *a* years was estimated as follows: $T_{m,y,a}$=1 if *a*+*y* < 2015, $T_{m,y,a}$=$\frac{(12-m+0.5)}{12}$ if *a*+*y*=2015, 0 otherwise.

Example: children born in June (*m*) of year 2011 (*y*)

$\mathrm{NB}_{june,2011}$=66,808

**Age 0 year:** their time at-risk will be $T_{june,2011,0}$=1 year, since 0+2011<2015 and the person-year, $\mathrm{PY}_{june,2011,0}$=66,808

**Age 1 year:** their time at-risk will be $T_{june,2011,1}$=1 year, since 1+2011<2015 and the person-year, $\mathrm{PY}_{june,2011,1}$=66,808

**Age 2 years:** their time at-risk will be $T_{june,2011,2}$=1 year, since 2+2011<2015 and the person-year, $\mathrm{PY}_{june,2011,2}$=66,808

**Age 3 years:** their time at-risk will be $T_{june,2011,3}$=1 year, since 3+2011<2015 and the person-year, $\mathrm{PY}_{june,2011,3}$=66,808

**Age 4 years:** their time at-risk will be $T_{june,2011,4}$=$\frac{(12-6+0.5)}{12}$=0.54 year, since 4+2011=2015 and the person-year, $\mathrm{PY}_{june,2011,4}$=66,808*0.54=36,187.7

**Age 5 to 14 years:** their time at-risk will be $T_{june,2011,a}=0$ year, since *a*+2011>2015 and the person-year, $\mathrm{PY}_{june,2011,a}$=0

# Appendix 2. Formula for calculation of age-specific person-years at risk for the analyses by month of diagnosis with an example

For the analyses by month of diagnosis, the age-specific person-years at risk were estimated as the average population of two consecutive years and weighted by the number of days in the month:

$\mathrm{PY}_{m,y,a}=\mathrm{PY}_{y,a}*\frac{\mathrm{ND}_{m,y}}{\mathrm{ND}_{.,y}}$, with $\mathrm{PY}_{y,a}=\frac{\mathrm{POP}_{y,a}+\mathrm{POP}_{(y+1),a}}{2}$

in which $\mathrm{PY}_{m,y,a}$ is the person-years for month *m* of year *y* and age *a* (*a*=0 to 14 years); $\mathrm{PY}_{y,a}$, the total person-years estimated for *y* and *a*; $\mathrm{ND}_{m,y}$, the number of days in month *m* of year *y*; $\mathrm{ND}_{.,y}$, the total number of days in year *y* (365 or 366 for leap years); $\mathrm{POP}_{y,a}$ and $\mathrm{POP}_{(y+1),a}$ are the numbers of children living in France and aged *a* years on 1st January of the years *y* and *y*+1, respectively.

Considering an example of children who lived in mainland France in 2011 (*y*) and were at risk of developing an embryonal tumour in June (*m*):

**Age 0 year:** $\mathrm{POP}_{2011,0}$=778,272; $\mathrm{POP}_{2012,0}$=763,531; $\mathrm{PY}_{2011,0}$=(778,272+763,531)/2=770,901.5 and, $\mathrm{PY}_{June,2011,0}$=770,901.5 *(30/365)=63,361.8

**Age 1 year:** $\mathrm{POP}_{2011,1}$=771,037; $\mathrm{POP}_{2012,1}$=781,550; $\mathrm{PY}_{2011,1}$=(771,037+781,550)/2=776,293.5 and, $\mathrm{PY}_{June,2011,1}$=776,293.5*(30/365)=63,804.9

**Age 2 years:** $\mathrm{POP}_{2011,2}$=773,987; $\mathrm{POP}_{2012,2}$=775,094; $\mathrm{PY}_{2011,2}$=(773,987+775,094)/2=774,540.5 and, $\mathrm{PY}_{June,2011,2}$=774,540.5*(30/365)=63,660.9

**Age 3 years:** $\mathrm{POP}_{2011,3}$=770,561; $\mathrm{POP}_{2012,3}$=780,356; $\mathrm{PY}_{2011,3}$=(770,561+780,356)/2=775,458.5 and, $\mathrm{PY}_{June,2011,3}$=775,458.5*(30/365)=63,736.3

**Age 4 years:** $\mathrm{POP}_{2011,4}$=787,730; $\mathrm{POP}_{2012,4}$=778,613; $\mathrm{PY}_{2011,4}$=(787,730+778,613)/2=783,171.5 and, $\mathrm{PY}_{June,2011,4}$=783,171.5*(30/365)=64,370.3

**Age 5 years:** $\mathrm{POP}_{2011,5}$=772,272; $\mathrm{POP}_{2012,5}$=794,066; $\mathrm{PY}_{2011,5}$=(772,272+794,066)/2=783,169 and, $\mathrm{PY}_{June,2011,5}$=783,169*(30/365)=64,370.1

**Age 6 years:** $\mathrm{POP}_{2011,6}$=770,076; $\mathrm{POP}_{2012,6}$=778,941; $\mathrm{PY}_{2011,6}$=(770,076+778,941)/2=774,508.5 and, $\mathrm{PY}_{June,2011,6}$=774,508.5*(30/365)=63,658.2

**Age 7 years:** $\mathrm{POP}_{2011,7}$=767,500; $\mathrm{POP}_{2012,7}$=775,211; $\mathrm{PY}_{2011,7}$=(767,500+775,211)/2=771,355.5 and, $\mathrm{PY}_{June,2011,7}$=771,355.5*(30/365)=63,399.1

**Age 8 years:** $\mathrm{POP}_{2011,8}$=774,875; $\mathrm{POP}_{2012,8}$=773,146; $\mathrm{PY}_{2011,8}$=(774,875+773,146)/2=774,010.5 and, $\mathrm{PY}_{June,2011,8}$=774,010.5*(30/365)=63,617.3

**Age 9 years:** $\mathrm{POP}_{2011,9}$=787,504; $\mathrm{POP}_{2012,9}$=779,378; $\mathrm{PY}_{2011,9}$=(787,504+779,378)/2=783,441 and, $\mathrm{PY}_{June,2011,9}$=783,441*(30/365)=64,392.4

**Age 10 years:** $\mathrm{POP}_{2011,10}$=804,235; $\mathrm{POP}_{2012,10}$=791,390; $\mathrm{PY}_{2011,10}$=(804,235+791,390)/2=797,812.5 and, $\mathrm{PY}_{June,2011,10}$=797,812.5*(30/365)=65,573.6

**Age 11 years:** $\mathrm{POP}_{2011,11}$=769,084; $\mathrm{POP}_{2012,11}$=809,104; $\mathrm{PY}_{2011,11}$=(769,084+809,104)/2=789,094 and, $\mathrm{PY}_{June,2011,11}$=789,094*(30/365)=64,857

**Age 12 years:** $\mathrm{POP}_{2011,12}$=764,508; $\mathrm{POP}_{2012,12}$=772,060; $\mathrm{PY}_{2011,12}$=(764,508+772,060)/2=768,284 and, $\mathrm{PY}_{June,2011,12}$=768,284*(30/365)=63,146.6

**Age 13 years:** $\mathrm{POP}_{2011,13}$=753,516; $\mathrm{POP}_{2012,13}$=768,042; $\mathrm{PY}_{2011,13}$=(753,516+768,042)/2=760,779 and, $\mathrm{PY}_{June,2011,13}$=760,779*(30/365)=62,529.8

**Age 14 years:** $\mathrm{POP}_{2011,14}$=763,947; $\mathrm{POP}_{2012,14}$=756,580; $\mathrm{PY}_{2011,14}$=(763,947+756,580)/2=760,263.5 and, $\mathrm{PY}_{June,2011,14}$=760,263.5*(30/365)=62,487.4

# Table S1. Variations in embryonal tumour incidence rates by year of birth – mainland France, 2000-2015 (source: RNCE)

| **Year of birth** | **Neuroblastoma (n=1,839)** | | | **Nephroblastoma (n=1,073)** | | | **Medulloblastoma (n=541)** | | | **Rhabdomyosarcoma (n=599)** | | | **Retinoblastoma (n=698)** | | | **Hepatoblastoma (n=206)** | | |
| --- | --- | --- | --- | --- | --- | --- | --- | --- | --- | --- | --- | --- | --- | --- | --- | --- | --- | --- |
|  | **O** | **E** | **SIR (95% CI)** | **O** | **E** | **SIR (95% CI)** | **O** | **E** | **SIR (95% CI)** | **O** | **E** | **SIR (95% CI)** | **O** | **E** | **SIR (95% CI)** | **O** | **E** | **SIR (95% CI)** |
| 2000 | 137 | 141.5 | 0.97 (0.82-1.14) | 83 | 95.1 | 0.87 (0.70-1.07) | 53 | 66.4 | 0.80 (0.61-1.05) | 53 | 62.4 | 0.85 (0.64-1.10) | 55 | 49.1 | 1.12 (0.85-1.44) | 9 | 15.4 | 0.58 (0.28-1.05) |
| 2001 | 124 | 139.5 | 0.89 (0.74-1.05) | 89 | 93.1 | 0.96 (0.77-1.17) | 50 | 63.7 | 0.79 (0.60-1.04) | 67 | 60.5 | 1.11 (0.86-1.39) | 49 | 48.8 | 1.00 (0.75-1.31) | 18 | 15.3 | 1.18 (0.71-1.81) |
| 2002 | 126 | 135.5 | 0.93 (0.78-1.10) | 82 | 89.1 | 0.92 (0.73-1.13) | 54 | 58.3 | 0.93 (0.71-1.21) | 52 | 56.6 | 0.92 (0.69-1.19) | 38 | 47.9 | 0.79 (0.57-1.07) | 14 | 14.9 | 0.94 (0.53-1.52) |
| 2003 | 140 | 133.0 | 1.05 (0.89-1.24) | 85 | 86.3 | 0.99 (0.79-1.21) | 46 | 53.7 | 0.86 (0.64-1.14) | 50 | 53.4 | 0.94 (0.70-1.22) | 53 | 47.7 | 1.11 (0.84-1.44) | 18 | 14.7 | 1.22 (0.74-1.88) |
| 2004 | 136 | 131.7 | 1.03 (0.87-1.22) | 102 | 84.1 | 1.21 (0.99-1.46) | 58 | 49.5 | 1.17 (0.91-1.52) | 61 | 50.7 | 1.20 (0.93-1.53) | 42 | 47.8 | 0.88 (0.64-1.17) | 7 | 14.7 | 0.48 (0.21-0.92) |
| 2005 | 129 | 130.4 | 0.99 (0.83-1.17) | 90 | 81.9 | 1.10 (0.89-1.34) | 58 | 45.2 | 1.28 (0.99-1.66) | 46 | 47.9 | 0.96 (0.71-1.26) | 50 | 48.0 | 1.04 (0.78-1.36) | 21 | 14.6 | 1.44 (0.91-2.14) |
| 2006 | 131 | 131.7 | 0.99 (0.83-1.17) | 91 | 81.3 | 1.12 (0.90-1.37) | 48 | 41.7 | 1.15 (0.87-1.53) | 52 | 46.0 | 1.13 (0.85-1.47) | 49 | 49.1 | 1.00 (0.74-1.30) | 13 | 14.8 | 0.88 (0.48-1.44) |
| 2007 | 147 | 127.4 | 1.15 (0.98-1.35) | 83 | 77.3 | 1.07 (0.86-1.32) | 45 | 36.4 | 1.24 (0.92-1.66) | 42 | 42.1 | 1.00 (0.72-1.33) | 70 | 48.2 | 1.45 (1.14-1.82) | 16 | 14.5 | 1.11 (0.65-1.74) |
| 2008 | 133 | 126.6 | 1.05 (0.88-1.24) | 74 | 75.3 | 0.98 (0.78-1.22) | 43 | 32.0 | 1.34 (1.00-1.81) | 44 | 39.4 | 1.12 (0.82-1.48) | 40 | 48.6 | 0.82 (0.59-1.11) | 18 | 14.4 | 1.25 (0.76-1.91) |
| 2009 | 131 | 123.6 | 1.06 (0.89-1.25) | 69 | 72.0 | 0.96 (0.75-1.20) | 22 | 27.1 | 0.81 (0.54-1.23) | 29 | 36.0 | 0.81 (0.55-1.14) | 40 | 48.1 | 0.83 (0.60-1.12) | 12 | 14.2 | 0.84 (0.45-1.42) |
| 2010 | 110 | 122.5 | 0.90 (0.74-1.08) | 80 | 69.9 | 1.15 (0.91-1.41) | 26 | 22.5 | 1.15 (0.79-1.70) | 29 | 33.1 | 0.88 (0.60-1.24) | 38 | 48.4 | 0.78 (0.56-1.06) | 11 | 14.2 | 0.78 (0.40-1.33) |
| 2011 | 112 | 114.5 | 0.98 (0.81-1.17) | 45 | 61.1 | 0.74 (0.54-0.97) | 23 | 17.7 | 1.30 (0.87-1.96) | 27 | 27.6 | 0.98 (0.66-1.40) | 44 | 46.3 | 0.95 (0.70-1.26) | 12 | 13.3 | 0.90 (0.48-1.51) |
| 2012 | 101 | 103.5 | 0.98 (0.80-1.18) | 49 | 48.0 | 1.02 (0.76-1.33) | 7 | 13.2 | 0.53 (0.25-1.11) | 24 | 20.6 | 1.17 (0.76-1.70) | 44 | 43.4 | 1.01 (0.74-1.34) | 15 | 12.1 | 1.24 (0.71-1.98) |
| 2013 | 90 | 87.0 | 1.03 (0.84-1.26) | 31 | 33.7 | 0.92 (0.63-1.28) | 8 | 8.4 | 0.95 (0.47-1.89) | 16 | 13.8 | 1.16 (0.68-1.83) | 40 | 38.2 | 1.05 (0.76-1.41) | 10 | 10.1 | 0.99 (0.50-1.74) |
| 2014 | 61 | 65.0 | 0.94 (0.72-1.19) | 17 | 19.1 | 0.89 (0.53-1.38) | 0 | 4.2 | - | 3 | 7.2 | 0.42 (0.10-1.08) | 33 | 28.0 | 1.18 (0.82-1.63) | 9 | 6.6 | 1.36 (0.65-2.46) |
| 2015 | 31 | 25.5 | 1.21 (0.83-1.69) | 3 | 5.8 | 0.52 (0.13-1.35) | 0 | 1.1 | - | 4 | 1.8 | 2.18 (0.68-5.07) | 13 | 10.3 | 1.27 (0.70-2.08) | 3 | 2.1 | 1.40 (0.35-3.63) |
| p |  |  | 0.84 |  |  | 0.35 |  |  | <0.01 |  |  | 0.53 |  |  | 0.17 |  |  | 0.38 |

95% CI, 95% Confidence Interval; n, total number of cases; O and E, yearly observed and expected number of cases, respectively; p, p-value of the likelihood ratio test for the overall heterogeneity of yearly SIRs; SIR, standardized incidence ratio

# Table S2. Variations in embryonal tumour incidence rates by year of diagnosis – mainland France, 2000-2015 (source: RNCE)

| **Year of diagnosis** | **Neuroblastoma (n=2,247)** | | | **Nephroblastoma (n=1,412)** | | | **Medulloblastoma (n=967)** | | | **Rhabdomyosarcoma (n=964)** | | | **Retinoblastoma (n=804)** | | | **Hepatoblastoma (n=241)** | | |
| --- | --- | --- | --- | --- | --- | --- | --- | --- | --- | --- | --- | --- | --- | --- | --- | --- | --- | --- |
|  | **O** | **E** | **SIR (95% CI)** | **O** | **E** | **SIR (95% CI)** | **O** | **E** | **SIR (95% CI)** | **O** | **E** | **SIR (95% CI)** | **O** | **E** | **SIR (95% CI)** | **O** | **E** | **SIR (95% CI)** |
| 2000 | 152 | 136.0 | 1.12 (0.95-1.30) | 88 | 85.0 | 1.04 (0.83-1.27) | 68 | 58.8 | 1.16 (0.90-1.45) | 46 | 58.3 | 0.79 (0.58-1.04) | 51 | 48.7 | 1.05 (0.79-1.36) | 11 | 14.6 | 0.76 (0.39-1.29) |
| 2001 | 123 | 137.9 | 0.89 (0.74-1.06) | 87 | 85.8 | 1.01 (0.82-1.24) | 64 | 58.9 | 1.09 (0.84-1.37) | 59 | 58.7 | 1.01 (0.77-1.28) | 54 | 49.5 | 1.09 (0.83-1.41) | 11 | 14.8 | 0.74 (0.39-1.28) |
| 2002 | 125 | 138.4 | 0.90 (0.75-1.07) | 82 | 86.5 | 0.95 (0.76-1.17) | 60 | 59.1 | 1.01 (0.78-1.29) | 61 | 59.0 | 1.03 (0.80-1.32) | 48 | 49.7 | 0.97 (0.72-1.27) | 11 | 14.9 | 0.74 (0.38-1.27) |
| 2003 | 155 | 138.4 | 1.12 (0.95-1.31) | 88 | 87.0 | 1.01 (0.81-1.24) | 54 | 59.3 | 0.91 (0.69-1.18) | 69 | 59.2 | 1.17 (0.91-1.46) | 48 | 49.6 | 0.97 (0.72-1.27) | 11 | 14.9 | 0.74 (0.38-1.27) |
| 2004 | 145 | 138.6 | 1.05 (0.88-1.23) | 84 | 87.3 | 0.96 (0.77-1.18) | 64 | 59.4 | 1.08 (0.84-1.36) | 53 | 59.3 | 0.89 (0.67-1.16) | 43 | 49.6 | 0.87 (0.63-1.15) | 17 | 14.9 | 1.14 (0.68-1.78) |
| 2005 | 130 | 139.0 | 0.94 (0.78-1.11) | 83 | 87.3 | 0.95 (0.76-1.17) | 59 | 59.4 | 0.99 (0.76-1.27) | 66 | 59.4 | 1.11 (0.86-1.40) | 48 | 49.7 | 0.97 (0.72-1.26) | 19 | 14.9 | 1.28 (0.78-1.94) |
| 2006 | 142 | 140.0 | 1.01 (0.86-1.19) | 86 | 87.5 | 0.98 (0.79-1.21) | 57 | 59.6 | 0.96 (0.73-1.23) | 57 | 59.6 | 0.96 (0.73-1.23) | 56 | 50.2 | 1.12 (0.85-1.43) | 18 | 15.0 | 1.20 (0.73-1.84) |
| 2007 | 122 | 140.9 | 0.87 (0.72-1.03) | 93 | 88.0 | 1.06 (0.86-1.29) | 53 | 59.9 | 0.88 (0.67-1.14) | 67 | 59.8 | 1.12 (0.87-1.41) | 49 | 50.6 | 0.97 (0.72-1.27) | 12 | 15.1 | 0.79 (0.43-1.33) |
| 2008 | 156 | 141.5 | 1.10 (0.94-1.28) | 95 | 88.6 | 1.07 (0.87-1.30) | 54 | 60.3 | 0.90 (0.68-1.16) | 59 | 60.2 | 0.98 (0.75-1.25) | 60 | 50.8 | 1.18 (0.91-1.51) | 16 | 15.2 | 1.05 (0.62-1.66) |
| 2009 | 153 | 142.4 | 1.07 (0.91-1.25) | 90 | 89.2 | 1.01 (0.81-1.23) | 59 | 60.8 | 0.97 (0.74-1.24) | 56 | 60.7 | 0.92 (0.70-1.19) | 50 | 51.0 | 0.98 (0.73-1.28) | 13 | 15.3 | 0.85 (0.47-1.40) |
| 2010 | 149 | 143.2 | 1.04 (0.88-1.22) | 84 | 89.8 | 0.94 (0.75-1.15) | 56 | 61.2 | 0.92 (0.70-1.18) | 64 | 61.1 | 1.05 (0.81-1.33) | 43 | 51.3 | 0.84 (0.61-1.11) | 17 | 15.4 | 1.11 (0.66-1.72) |
| 2011 | 130 | 143.5 | 0.91 (0.76-1.07) | 95 | 90.1 | 1.05 (0.86-1.28) | 68 | 61.5 | 1.11 (0.86-1.39) | 63 | 61.4 | 1.03 (0.81-1.33) | 42 | 51.4 | 0.82 (0.59-1.09) | 14 | 15.4 | 0.91 (0.51-1.47) |
| 2012 | 162 | 143.1 | 1.13 (0.97-1.32) | 96 | 90.3 | 1.06 (0.86-1.29) | 63 | 61.9 | 1.02 (0.79-1.29) | 52 | 61.7 | 0.84 (0.63-1.09) | 53 | 51.1 | 1.04 (0.78-1.34) | 19 | 15.4 | 1.24 (0.76-1.88) |
| 2013 | 134 | 142.3 | 0.94 (0.79-1.11) | 100 | 90.2 | 1.11 (0.91-1.34) | 58 | 62.2 | 0.93 (0.71-1.19) | 65 | 61.9 | 1.05 (0.82-1.33) | 50 | 50.7 | 0.99 (0.74-1.29) | 15 | 15.3 | 0.98 (0.57-1.57) |
| 2014 | 136 | 141.5 | 0.96 (0.81-1.13) | 80 | 89.9 | 0.89 (0.71-1.10) | 56 | 62.4 | 0.90 (0.68-1.15) | 65 | 61.9 | 1.05 (0.81-1.33) | 50 | 50.3 | 0.99 (0.74-1.29) | 15 | 15.2 | 0.99 (0.57-1.58) |
| 2015 | 133 | 140.4 | 0.95 (0.80-1.12) | 81 | 89.4 | 0.91 (0.72-1.12) | 74 | 62.4 | 1.19 (0.94-1.48) | 62 | 61.8 | 1.00 (0.77-1.27) | 59 | 49.9 | 1.18 (0.91-1.51) | 22 | 15.1 | 1.46 (0.93-2.16) |
| p |  |  | 0.29 |  |  | 0.99 |  |  | 0.90 |  |  | 0.86 |  |  | 0.89 |  |  | 0.74 |

95% CI, 95% Confidence Interval; n, total number of cases; O and E, yearly observed and expected number of cases, respectively; p, p-value of the likelihood ratio test for the overall heterogeneity of yearly SIRs; SIR, standardized incidence ratio

# Table S3. Distribution of neuroblastoma cases by month of birth and month of diagnosis by *MYCN* status (known vs. unknown) - mainland France, 2000-2015 (source: RNCE)

| **Month** | **Analysis by month of birth** | |  | **Analysis by month of diagnosis** | |
| --- | --- | --- | --- | --- | --- |
|  | **known *MYCN* status** | **unknown *MYCN* status** |  | **known *MYCN* status** | **unknown *MYCN* status** |
|  | **n=1,609 (87.5%)** | **n=230 (12.5%)** |  | **n=1,915 (85.2%)** | **n=332 (14.8%)** |
| January | 146 (88.5%) | 19 (11.5%) |  | 165 (85.9%) | 27 (14.1%) |
| February | 129 (94.2%) | 8 (5.8%) |  | 169 (87.6%) | 24 (12.4%) |
| March | 139 (88.5%) | 18 (11.5%) |  | 160 (85.1%) | 28 (14.9%) |
| April | 117 (82.4%) | 25 (17.6%) |  | 161 (86.1%) | 26 (13.9%) |
| May | 139 (88.0%) | 19 (12.0%) |  | 170 (86.7%) | 26 (13.3%) |
| June | 148 (90.8%) | 15 (9.2%) |  | 157 (80.5%) | 38 (19.5%) |
| July | 127 (88.8%) | 16 (11.2%) |  | 161 (86.1%) | 26 (13.9%) |
| August | 133 (89.3%) | 16 (10.7%) |  | 152 (85.9%) | 25 (14.1%) |
| September | 125 (85.0%) | 22 (15.0%) |  | 150 (84.7%) | 27 (15.3%) |
| October | 157 (84.0%) | 30 (16.0%) |  | 151 (85.8%) | 25 (14.2%) |
| November | 120 (82.8%) | 25 (17.2%) |  | 164 (85.9%) | 27 (14.1%) |
| December | 129 (88.4%) | 17 (11.6%) |  | 155 (82.4%) | 33 (17.6%) |
| p | 0.37 | |  | 0.99 | |

n, total number of cases; p, p-value of χ^2^ test of homogeneity

# Table S4. Distribution of the embryonal tumour cases included in the analyses by month of birth, and by diagnostic group, gender and age - mainland France, 2000-2015 (source: RNCE)

|  |  | **Total** |  |  | **Gender** | | | | |  | **Age at diagnosis** | | | | | | | | | |
| --- | --- | --- | --- | --- | --- | --- | --- | --- | --- | --- | --- | --- | --- | --- | --- | --- | --- | --- | --- | --- |
|  |  |  |  |  | **boys** | | **girls** | | **sex ratio** |  | **< 1 year** | | **1 year** | | **2 years** | | **3-4 years** | | **≥ 5 years** | |
| **Diagnosis** | **n** | **%** | **IR** |  | **n** | **%** | **n** | **%** |  |  | **n** | **%** | **n** | **%** | **n** | **%** | **n** | **%** | **n** | **%** |
| **Neuroblastoma** | 1,839 | *37.1* | 18.6 |  | 993 | *54.0* | 846 | *46.0* | 1.1 |  | 823 | *44.8* | 348 | *18.9* | 271 | *14.7* | 251 | *13.6* | 146 | *7.9* |
| localized | 996 | *54.2* | 10.1 |  | 533 | *53.5* | 463 | *46.5* | 1.1 |  | 516 | *51.8* | 201 | *20.2* | 106 | *10.6* | 100 | *10.0* | 73 | *7.3* |
| metastatic | 843 | *45.8* | 8.5 |  | 460 | *54.6* | 383 | *45.4* | 1.1 |  | 307 | *36.4* | 147 | *17.4* | 165 | *19.6* | 151 | *17.9* | 73 | *8.7* |
| *MYCN* not amplified | 1,307 | *71.1* | 13.2 |  | 675 | *51.6* | 632 | *48.4* | 1.0 |  | 644 | *49.3* | 207 | *15.8* | 170 | *13.0* | 182 | *13.9* | 104 | *8.0* |
| *MYCN* amplified | 302 | *16.4* | 3.1 |  | 186 | *61.6* | 116 | *38.4* | 1.5 |  | 57 | *18.9* | 101 | *33.4* | 80 | *26.5* | 43 | *14.2* | 21 | *7.0* |
| Unknown *MYCN* status | 230 | *12.5* | 2.3 |  | 132 | *57.4* | 98 | *42.6* | 1.3 |  | 122 | *53.0* | 40 | *17.4* | 21 | *9.1* | 26 | *11.3* | 21 | *9.1* |
| **Nephroblastoma** | 1,073 | *21.6* | 10.8 |  | 504 | *47.0* | 569 | *53.0* | 0.8 |  | 186 | *17.3* | 208 | *19.4* | 201 | *18.7* | 304 | *28.3* | 174 | *16.2* |
| **Medulloblastoma** | 541 | *10.9* | 5.5 |  | 356 | *65.8* | 185 | *34.2* | 1.8 |  | 37 | *6.8* | 53 | *9.8* | 66 | *12.2* | 104 | *19.2* | 281 | *51.9* |
| **Rhabdomyosarcoma** | 599 | *12.1* | 6.1 |  | 364 | *60.8* | 235 | *39.2* | 1.5 |  | 59 | *9.8* | 99 | *16.5* | 86 | *14.4* | 163 | *27.2* | 192 | *32.1* |
| **Retinoblastoma** | 698 | *14.1* | 7.1 |  | 363 | *52.0* | 335 | *48.0* | 1.0 |  | 331 | *47.4* | 195 | *27.9* | 92 | *13.2* | 65 | *9.3* | 15 | *2.1* |
| unilateral | 468 | *67.0* | 4.7 |  | 233 | *49.8* | 235 | *50.2* | 0.9 |  | 176 | *37.7* | 136 | *29.1* | 84 | *18.0* | 60 | *12.8* | 12 | *2.6* |
| bilateral | 230 | *33.0* | 2.3 |  | 130 | *56.5* | 100 | *43.5* | 1.2 |  | 155 | *67.4* | 59 | *25.7* | 8 | *3.5* | 5 | *2.2* | 3 | *1.3* |
| **Hepatoblastoma** | 206 | *4.2* | 2.1 |  | 131 | *63.6* | 75 | *36.4* | 1.7 |  | 69 | *33.5* | 63 | *30.6* | 35 | *17.0* | 28 | *13.6* | 11 | *5.3* |
| **All embryonal tumours** | 4,956 | *100* | 50.1 |  | 2,711 | *54.7* | 2,245 | *45.3* | 1.2 |  | 1,505 | *30.4* | 966 | *19.5* | 751 | *15.2* | 915 | *18.5* | 819 | *16.5* |

IR, annual incidence rate (cases/million/year); n, total number of cases; sex ratio, male to female IR ratio

# Table S5. Monthly variations based on twelve 30-day non-calendar month periods - mainland France, 2000-2015 (source: RNCE)

| **Month of birth** | **Rhabdomyosarcoma in boys (n=361)** | | | |  | **Month of diagnosis** | **Unilateral retinoblastoma (n=552)** | | | |
| --- | --- | --- | --- | --- | --- | --- | --- | --- | --- | --- |
|  | **O** | **E** | **SIR (95% CI)** | **p_H_** |  |  | **O** | **E** | **SIR (95% CI)** | **p_H_** |
| 15 January - 14 February | 21 | 30.1 | 0.70 (0.44-1.04) | 0.09 |  | 15 January - 14 February | 49 | 47.1 | 1.04 (0.78-1.36) | 0.10 |
| 15 February - 14 March | 19 | 29.6 | 0.64 (0.39-0.97) |  |  | 15 February - 14 March | 41 | 42.9 | 0.96 (0.69-1.28) |  |
| 15 March - 14 April | 26 | 30.2 | 0.86 (0.57-1.24) |  |  | 15 March - 14 April | 45 | 47.1 | 0.96 (0.70-1.26) |  |
| 15 April - 14 May | 33 | 30.5 | 1.08 (0.75-1.49) |  |  | 15 April - 14 May | 57 | 45.6 | 1.25 (0.95-1.60) |  |
| 15 May - 14 June | 40 | 30.6 | 1.31 (0.94-1.75) |  |  | 15 May - 14 June | 42 | 47.1 | 0.89 (0.65-1.19) |  |
| 15 June - 14 July | 29 | 31.0 | 0.94 (0.64-1.32) |  |  | 15 June - 14 July | 36 | 45.6 | 0.79 (0.56-1.08) |  |
| 15 July - 14 August | 32 | 31.3 | 1.02 (0.71-1.42) |  |  | 15 July - 14 August | 36 | 47.1 | 0.76 (0.54-1.04) |  |
| 15 August - 14 September | 45 | 30.4 | 1.48 (1.09-1.96) |  |  | 15 August - 14 September | 35 | 47.1 | 0.74 (0.52-1.02) |  |
| 15 September -14 October | 30 | 30.2 | 0.99 (0.68-1.39) |  |  | 15 September -14 October | 48 | 45.6 | 1.05 (0.78-1.38) |  |
| 15 October - 14 November | 28 | 29.4 | 0.95 (0.64-1.35) |  |  | 15 October - 14 November | 60 | 47.1 | 1.27 (0.98-1.62) |  |
| 15 November - 14 December | 32 | 28.8 | 1.11 (0.77-1.54) |  |  | 15 November - 14 December | 51 | 45.6 | 1.12 (0.84-1.45) |  |
| 15 December - 14 January | 26 | 28.8 | 0.90 (0.60-1.30) |  |  | 15 December - 14 January | 52 | 44.2 | 1.18 (0.89-1.53) |  |
|  |  |  |  |  |  |  |  |  |  |  |
| **Temporal window detected by SaTScan™** | | |  | **p** |  | **Temporal cluster detection by SaTScan™** | | |  | **p** |
| 15 December - 14 April | 92 | 118.7 | 0.78 (0.62-0.95) | 0.08 |  | 15 May - 14 September | 149 | 186.9 | 0.80 (0.67-0.94) | 0.01 |
| 15 April - 14 December | 269 | 242.3 | 1.11 (0.98-1.25) |  |  | 15 September - 14 May | 403 | 365.1 | 1.10 (1.00-1.22) |  |

95% CI, 95% Confidence Interval; n, total number of cases; O and E, monthly observed and expected number of cases; p_H_, statistical significance threshold (p-value) of the likelihood ratio test for the overall monthly heterogeneity; p, p-value for the most likely cluster estimated with 9,999 Monte Carlo simulations (SaTScan™); SIR, standardized incidence ratio

# Table S6. Variations in medulloblastoma incidence rates by month of birth using annual age-specific incidence rates as reference rates - mainland France, 2000-2015 (source: RNCE)

| **Month of birth** | **Medulloblastoma (n=541)** | | | |
| --- | --- | --- | --- | --- |
|  | **O** | **E** | **SIR (95% CI)** | **p_H_** |
| January | 45 | 47.9 | 0.94 (0.69-1.24) | 0.73 |
| February | 45 | 43.3 | 1.04 (0.77-1.37) |  |
| March | 38 | 46.0 | 0.83 (0.59-1.12) |  |
| April | 36 | 44.7 | 0.81 (0.57-1.10) |  |
| May | 52 | 46.7 | 1.11 (0.84-1.44) |  |
| June | 37 | 44.7 | 0.83 (0.59-1.12) |  |
| July | 49 | 47.4 | 1.03 (0.77-1.35) |  |
| August | 51 | 45.8 | 1.11 (0.84-1.45) |  |
| September | 48 | 44.5 | 1.08 (0.80-1.41) |  |
| October | 44 | 45.0 | 0.98 (0.72-1.30) |  |
| November | 47 | 42.2 | 1.11 (0.83-1.46) |  |
| December | 49 | 42.8 | 1.15 (0.85-1.50) |  |

95% CI, 95% Confidence Interval; n, total number of cases; O and E, monthly observed and expected number of cases; p_H_, statistical significance threshold (p-value) of the likelihood ratio test for the overall monthly heterogeneity; SIR, standardized incidence ratio
